# Critical-Size Muscle Defect Regeneration Using an Injectable Cell-Laden Nanofibrous Matrix: An Ex Vivo Mouse Hindlimb Organ Culture Study

**DOI:** 10.3390/ijms262412120

**Published:** 2025-12-17

**Authors:** Diego Jacho, James Huynh, Emily Crowe, Agustin Rabino, Mine Yıldırım, Piotr J. Czernik, Beata Lecka-Czernik, Rafael Garcia-Mata, Eda Yildirim-Ayan

**Affiliations:** 1 Department of Bioengineering, College of Engineering, University of Toledo, Toledo, OH 43606, USAjames.huynh@rockets.utoledo.edu (J.H.); 2 Department of Orthopedic Surgery, College of Medicine and Life Sciences, University of Toledo, Toledo, OH 43606, USA; emily.crowe@rockets.utoledo.edu (E.C.); piotr.czernik@utoledo.edu (P.J.C.); beata.leckaczernik@utoledo.edu (B.L.-C.); 3 Department of Biological Science, University of Toledo, Toledo, OH 43606, USArafael.garciamata@utoledo.edu (R.G.-M.); 4 Department of Common Courses, Kadir Has University, Istanbul 34083, Turkey; mine.yildirim@khas.edu.tr; 5 Center for Diabetes and Endocrine Research, University of Toledo, Toledo, OH 43606, USA

**Keywords:** 3D tissue culturing, muscle, tibialis, defect, regeneration, collagen, tissue, engineering, organ, culture, ex vivo, mechanical loading, animal welfare, mechanotherapy

## Abstract

Musculoskeletal injuries involving volumetric muscle loss remain difficult to treat due to limited regenerative capacity and the lack of physiologically relevant experimental models. This study introduces a computer-controlled ex vivo mouse hindlimb culturing platform that applies dynamic mechanical loading to evaluate muscle regeneration in a critical-size tibialis anterior (TA) defect. The defect was treated with an injectable myoblast-laden nanofibrous scaffold composed of polycaprolactone nanofibers and collagen (PNCOL). The ex vivo mouse hindlimb culturing platform maintained tissue viability and transmitted physiological strain across bone and muscle without disrupting the unity of the bone–muscle structure. PNCOL treatment under mechanical loading enhanced muscle fiber organization, extracellular matrix regeneration, and anti-inflammatory responses (CD206) while upregulating *paired box 7 (PAX7)*, *myogenic factor 5 (MYF5)*, *myogenic regulatory factor 4 (MRF4)*, *and transforming growth factor beta1 (TGFβ1)* expression. Cytokine profiling revealed an anabolic shift involving wingless/integrated (WNT) and insulin-like growth factor-1 (IGF-1) signaling, indicating a pro-regenerative microenvironment. Overall, the combination of mechanical stimulation and biomaterial-based therapy significantly improved muscle regeneration within a controlled ex vivo model. This multidimensional approach provides a reproducible and ethical platform that advances musculoskeletal regenerative research while reducing animal use.

## 1. Introduction

Musculoskeletal (MSK) tissue disorders are among the most prevalent physiological diseases worldwide, affecting approximately 1.71 billion people [1,2,3,4]. The exploration of novel methodologies specifically through cell therapy and mechanical loading in forms of mechanotherapy [5] holds promise for enhancing MSK tissue regeneration. There has been an increase in autologous mesenchymal stem cell (MSC) utilization for various MSK injury treatments [6,7,8]. To date, bone marrow-derived MSCs are the most frequently used cell source for MSK tissue repair by delivering them to the area of interest via a syringe or other minimally invasive techniques [9,10]. However, the injection of MSCs into the defect area comes with several challenges including post-injection cell death [9,10] due to the harsh immune environment at the defect site and possible migration of the MSCs to the other areas, subsequently creating ectopic tissue formations. To prevent these challenges, the MSCs are encapsulated within a synthetic and/or natural matrix, which serves as a temporary home for cells until tissue regeneration and as a cell barrier matrix for harsh external environments [10,11,12].

Utilizing synthetic and/or natural matrix along with cell therapy is particularly important for critical-sized defects with a size ranging from 1 to 3 cm [13] and volumetric muscle loss (VML) [14]. The natural tissue repair mechanism is overwhelmed in regenerating large tissue structures for critical-sized bone defects and VLM cases. To address this, cellular or acellular synthetic and/or natural matrices are designed as filler materials, not only for carrying and protecting the encapsulated cells, serving as an inductive template to recruit endogenous stem/progenitor cells to injury sites for repair [15,16,17], but also for increasing mechanical stability at the defect site [8]. For instance, Zhang Z. et al. [9] designed conductive porous nanocomposite three-dimensional (3D) microgels loaded with myoblast, which significantly improved the generation of new muscle fibers, blood vessels, and anti-inflammatory properties at a mouse VML injury site. Numerous seminal studies discuss the design criterion of synthetic and/or natural matrices and their synthesis process for bone and muscle regeneration [18,19,20,21]; however, their performances in functional bone and muscle regenerations have yielded highly variable results [22,23,24,25] with limited in vivo application success.

The translation of in vitro success to in vivo applications is often hindered because many studies are conducted on two-dimensional (2D) surfaces (well plate and Petri dishes) without accounting for the surrounding extracellular matrix (ECM) and/or the dynamic mechanical environment of bones and muscles during culturing, which are crucial for functional muscle and bone regeneration and tissue recoveries [26]. To address this gap, in vivo studies have been conducted to take advantage of the cell–matrix interaction within the 3D environment under physiological mechanical loading conditions. For instance, Kim J. et al. [27] explored tibialis anterior (TA) VML injuries in a rat model using allogenic decellularized skeletal muscle scaffolds, revealing the age, stiffness, and size of scaffolds as obstacles to regenerative medicine strategies targeting VML injury. Similarly, Sicari B. et al. [24] used an in vivo VML model to assess the efficacy of surgically placed inductive biologic scaffold material on mouse quadriceps, highlighting the therapeutic potential of a reproducible animal model to study VML. While in vivo studies have numerous superiorities over in vitro studies, they have their limitations as well, including not being cost-efficient and being inherently inconsistent due to the biological variability and complexity of the animals [28,29]. In addition, variability in post-defect behavior among animals, where some may exhibit normal locomotion while others show limited movement or inactivity, can lead to inconsistent results, particularly in studies focusing on musculoskeletal tissues [30,31,32].

To circumvent these challenges, ex vivo MSK tissue culturing platforms with physiologically relevant mechanical stimulation have emerged. The ex vivo platforms offer a powerful tool for dissecting the specific mechanisms that drive tissue regeneration. The ex vivo models isolate and control specific variables and eliminate certain in vivo confounding factors (such as animal-to-animal differences in behavior or systemic responses) and allow for high control over mechanical and biochemical conditions. However, it should be noted that ex vivo models are not a replacement for in vivo studies and cannot capture systemic interactions. The in vivo models still remain valuable for studying whole-organism physiology. The ex vivo methods enable researchers to obtain clearer insights into the underlying mechanisms of tissue response and regeneration. This reduces confounding variables and extraneous factors that may influence results in vivo, leading to a clearer understanding of the underlying mechanisms at play. The ex vivo studies mitigate the complexities and ethical concerns associated with in vivo studies, aligning with the 4Rs principle (Reduction, Refinement, Replacement and Responsibility) [33]. In sum, compared to in vivo studies, ex vivo models can replace certain in vivo studies, reduce the number of animals required, and refine the methods to minimize animal suffering and improve animal welfare, and help to deliver faster, more reproducible, cost-effective, and responsible results [34,35,36].

In addition, ex vivo models, unlike 2D studies, preserve crucial 3D cell–cell and cell–matrix interactions, enabling the examination of native cells and tissues within their natural physiological environment under physiological mechanical loading with a fraction of the cost of in vivo studies and with higher consistency [37,38,39,40,41]. For instance, Passipieri J. et al. [6,42] developed an implantable tissue-engineered construct that facilitated substantial recovery in a VML injury model using ex vivo muscle extracted from rodents. Similarly, Sargent M. et al. [43] used muscle tissue from Sprague Dawley rats to study the response to physical stress, highlighting the efficacy of ex vivo models under mechanical stimulation in understanding the severity of muscle damage in MSK injuries. These ex- vivo studies and many others shed light on how bone and muscle regenerate under mechanical loading using various interventions (cell therapy, biomaterial-based therapies, etc.); however, they isolate either bone or muscle based on their interest and only focus on one tissue during ex vivo culturing [43,44,45,46]. Whole ex vivo organ models, encompassing both bone and muscle, can offer a unique advantage over single-tissue studies [39] by capturing the intricate interplay between bone and muscle physiology, thereby providing a more relevant understanding of MSK disorders.

Towards this end, in this study, a computer-controlled ex vivo mouse hindlimb culturing platform operating under dynamic mechanical loading was developed to study the effect of cell-laden nanofibrous scaffold, PNCOL, on a critical-size defect on tibialis anterior (TA) muscle regeneration. PNCOL is a proprietary nanofibrous composite matrix composed of electrospun polycaprolactone (PCL) nanofibers dispersed within a type-I collagen gel [47]. In PNCOL, the collagen provides a biomimetic extracellular matrix that supports cell adhesion, viability, and matrix remodeling, while the embedded PCL nanofibers provide structural integrity and mechanical strength. Cells either alone or co-delivered with growth factors can be encapsulated within the PNCOL scaffold, where they secrete native extracellular matrix proteins and cytokines that contribute to the regenerative microenvironment. This synergistic design results in an injectable, cell- and biologics-laden scaffold capable of mimicking native tissue architecture while being delivered in a minimally invasive manner. Due to its tunable composition and compatibility with soft and load-bearing tissues, PNCOL has been successfully applied in regenerative models including bone [15,48] and annulus fibrosus [49,50] using both 3D in vitro platforms and ex vivo organ culture systems.

The ex vivo mouse hindlimbs with TA muscles defects were treated with and without PNCOL and then were cultured for 7 days under 12% strain with 0.1 Hz frequency. Following comprehensive histological, molecular, and proteomic analyses, it was proved that the ex vivo mouse hindlimb culturing platform maintained the physiological functions of the entire hindlimbs, and the TA defect treated with PNCOL demonstrated better regeneration than the non-treated defect under dynamic mechanical loading conditions.

## 2. Results

### 2.1. Ex Vivo Organ Culture Platform Efficiency on Whole Mouse Hindlimbs

**Figure 1:**
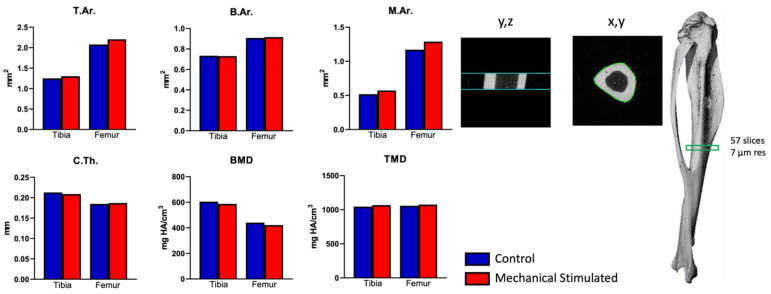
Micro-CT analysis of midshaft femur at the end of experiment. Measurements: total area (bone plus marrow area) (T.Ar.), bone area (B.Ar.), marrow area (M.Ar.), mean cortical thickness (C.Th.), bone mineral density (BMD), and tissue mineral density (TMD).

First, our examination of the whole mouse hindlimb within our organ culture provided significant insights into the effects of mechanical stimulation on bone structure. Utilizing micro-CT analysis, we focused on the bone area of the stimulated samples, particularly emphasizing mechanical loading stimulation. Our results, as presented in Figure 1, highlight changes in the tibia and femur midshaft cortical bone after stimulation. Following the stimulation, both the tibia and femur exhibited notable increases in total bone and marrow area compared to the control group (unstimulated). Total midshaft bone area increased in stimulated femur and tibia by 6% and 4%, respectively, while cortical thickness and bone area remained rather unchanged (±1%). However, the marrow area increased by 10% in both bones. These changes suggest a mild bone adaptive response to mechanical loading. Overall, these results indicate that within the duration of the experiment, bone tissue was alive and responded to the biomechanical stimulation and altered environment.

### 2.2. Cell Viability Within the Mouse Hindlimb After Treatments

After demonstrating the efficiency of the organ culture platform in applying mechanical stimulation to the entire mouse hindlimbs, 2 mm damage sites were created at the TA muscle and treated with myoblast-encapsulated polycaprolactone and collagen scaffolds, PNCOL (+). The damage sites without scaffolds, PNCOL (−), were used as control.

**Figure 2:**
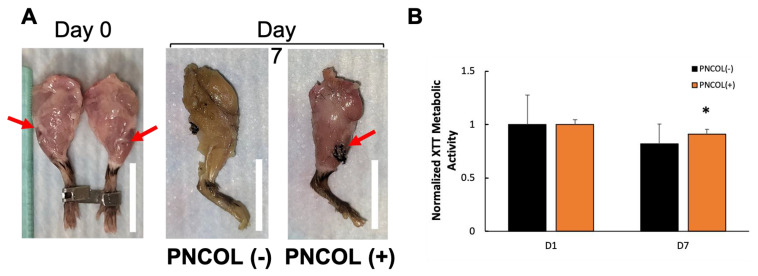
(**A**) Representative optical images of PNCOL (−) and PNCOL (+) mouse hindlimbs after mechanical stimulation. Scale bar = 20 mm, red arrow demonstrates damage site, and (**B**) cellular metabolic activity at days 1 and 7 after treatment and mechanical stimulation (n = 4). (*) indicates the statistical differences between control and experimental groups with *p* < 0.05.

Gross optical images revealed notable morphological contrasts between the hindlimbs treated with PNCOL and their untreated counterparts (Figure 2A). The PNCOL (+) group exhibited a fully restored, robust skeletal muscle structure with normal coloration. Conversely, in the control group, PNCOL (−), the puncture defect remained visible after 7 days of organ culturing. The cell viability was measured throughout the whole organ culture (7 days) period using the colorimetric-XTT assay. Figure 2B demonstrates that the cell viability was preserved throughout the organ culture period for both PNCOL (−) and PNCOL (+) samples. These results demonstrated that the whole organ culture was successfully kept viable in culture during the period of the study.

### 2.3. Structural and Morphological Stability of TA Muscle Defect Site After Treatments

**Figure 3:**
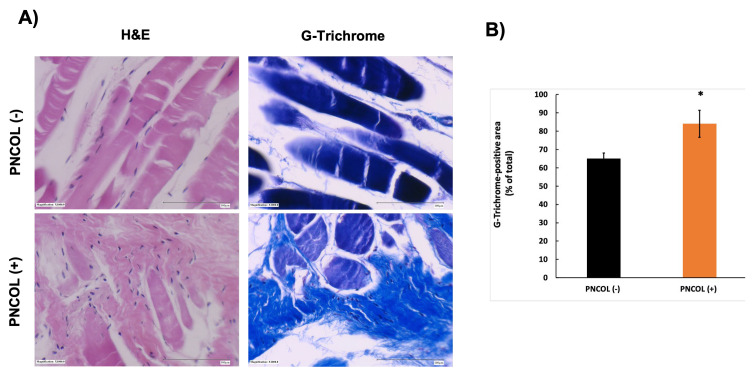
(**A**) Representative histological images of the TA muscle stained with H&E and G-trichrome after 7 days of PNCOL treatment and mechanical stimulation (1000×). Scale bar = 100 µm. (**B**) Quantitative analysis of G-trichrome-positive areas relative to the total tissue area for the PNCOL (−) and PNCOL (+) groups. Bars represent mean ± SD (n = 4). * indicates significant difference compared with control (* *p* < 0.05).

To further explore the effects of PNCOL treatment and mechanical stimulation on TA muscle healing after damage, we conducted a histological analysis of the damage site. In the PNCOL (−) group, Figure 3 histology images revealed a notable absence of tissue regeneration and cell density. PNCOL (−) samples exhibited more space within the damaged area, resulting in no observable regeneration after 7 days of organ culturing. In contrast, the PNCOL (+) group displayed a higher number of cell nuclei in H&E staining.

Additionally, the slides were stained with G-trichrome to evaluate collagen and extracellular matrix (ECM) organization within the defect region. In the PNCOL (−) group, there were noticeable gaps within the defect site, indicating limited matrix regeneration. By contrast, the PNCOL (+) group presented denser collagen bundles that may suggest more robust ECM remodeling and structural repair. The quantitative image analysis of G-trichrome histological slides showed the G-trichrome-positive area between the PNCOL (–) and PNCOL (+) groups. The G-trichrome-positive area, which reflects collagen fiber content and extracellular matrix organization, increased from 68 ± 6.5% in the PNCOL (–) group to 88 ± 4.2% in the PNCOL (+) group (*p* < 0.05). The quantitative data confirm that PNCOL treatment substantially enhances extracellular matrix remodeling within the critical-sized TA defect after 7 days of healing.

**Figure 4:**
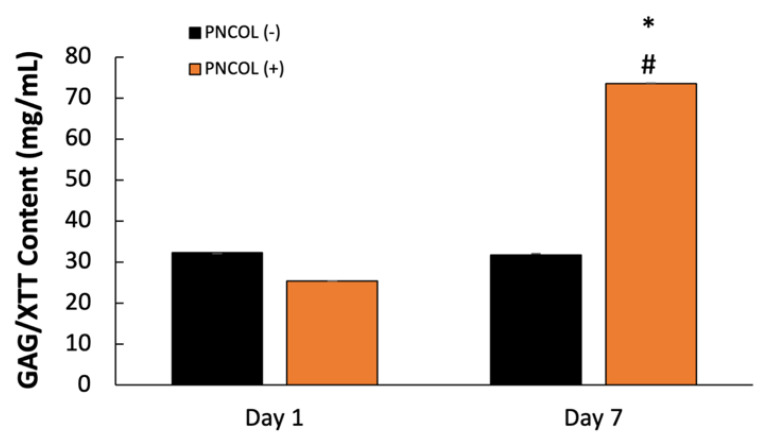
Proteoglycans production. GAG/XTT (mg/mL) content at days 1 and 7 after PNCOL treatment and mechanical stimulation (n = 4). (*) indicates the statistical differences between control and experimental groups with *p* < 0.05; (#) indicates a statistical difference between experimental groups of the same-day data point.

Injury recovery hinges on a balance of proteoglycan production; excessive levels can lead to scarring and tissue damage. Figure 4 illustrates heightened proteoglycan production in the PNCOL (+) group, indicative of robust muscle recovery and tissue regeneration, likely influenced by mechanical stimulation and PNCOL treatment. Conversely, the control group exhibited no significant regeneration in the defect area.

### 2.4. Matrix Organization and Muscle Fiber Alignment of TA Muscle Defect Site After Treatments

**Figure 5:**
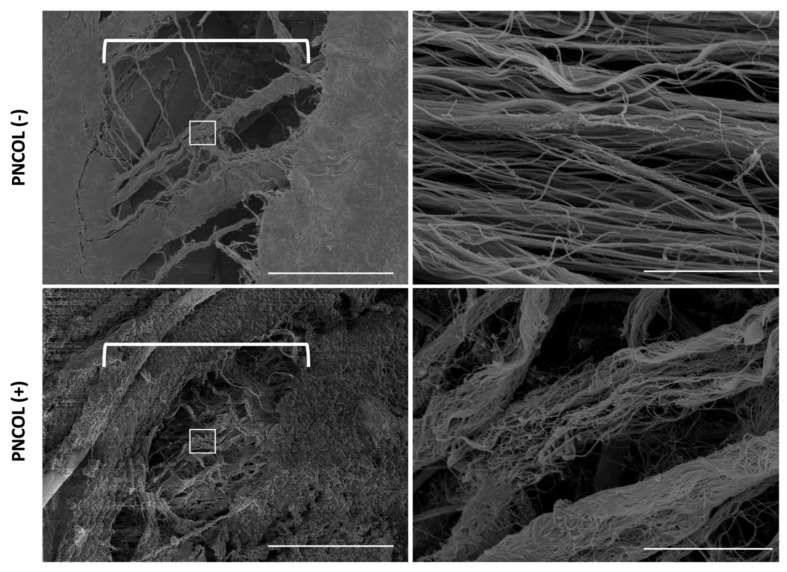
Representative scanning electron micrographs of PNCOL (−) and PNCOL (+) damage sites after 7 days of mechanical stimulation. The section under the brackets represents the damaged site area. Graphs on the right panel represent the zoom section of the damaged site. Scale bar is 50 µm for the left panel and 10 µm for the right panel (n = 4).

The representative SEM images of the TA defect treated with PNCOL and not treated are in Figure 5. The SEM images show that the defect area without any treatment highlighted by white brackets, exhibits a clear gap, while the defect area treated with PNCOL, PNCOL (+) group, demonstrates ECM production at the defect area. The PNCOL (+) SEM images showed more robust, compact fibers of muscle along the damage site, while the PNCOL (−) group showed thin and wispy ECM projections between the interwoven meshes of collagen and tissue.

### 2.5. Cytokine and Chemokine Expression of Mouse Hindlimb Medium After Treatments

**Figure 6:**
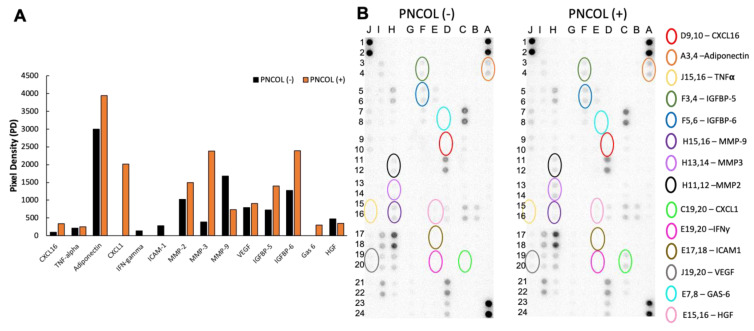
Cytokine levels in conditioned media (CM) collected from PNCOL (+) and PNCOL (−) samples on day 7. (**A**) Differentially secreted high- and low-abundance cytokines under PNCOL (−) (black bars) and PNCOL (+) (orange bars) conditions. (**B**) Membranes of the proteome cytokine arrays incubated with CM from PNCOL (+) and PNCOL (−) samples with encircled spots corresponding to a high abundance of differentially expressed cytokines.

To investigate the effect of PNCOL and mechanical stimulation on the mouse hindlimb culture after treatments, we used proteome profiler arrays coated specifically with antibodies against mouse cytokines and chemokines. Media from both groups were collected and analyzed after 7 days of treatment. Several cytokines and chemokines were released to the media in response to both treatments (Figure 6). Among them are factors involved in muscle regeneration (Adiponectin), inflammation and wound healing (CXCL1 and CXCL16), tissue remodeling (MMP2 and MMP3), and proteins that modulate IGF-1 signaling (IGFBP5 and IGFBP6). Consistent with bone response to both mechanical stimulation and PNCOL treatment, the levels of osteopontin and osteoprotegerin were increased. On the other hand, there was a decrease in the level of two inhibitors of the WNT signaling pathway (DKK1 and WISP1), the pathway which is anabolic for muscle and bone. Simultaneously, levels of several cytokines involved in inflammation, markers for chronic inflammation, and pro-inflammatory responses like ICAM-1, IFN-γ, and MMP9 were decreased in the PNCOL (+) group. Together, the cytokine profile suggests a dynamic remodeling of the ECM, which is essential for tissue repair and regeneration, and is supported by the muscle and bone secretome. This aligns with the overall findings of our study, which demonstrate a modulation of immune responses and a favorable shift towards enhanced muscle regeneration in the PNCOL (+) group compared to the untreated counterparts. On the other hand, the cytokines and chemokines involved in cell regulation, proliferation, differentiation, and tissue remodeling were upregulated in the PNCOL (+) group compared to the PNCOL (−) group. Because detecting proteins secreted at a low level and diluted in the culture medium is difficult, we confirmed the major markers by RT-qPCR.

### 2.6. Gene Expression Analysis of TA Muscle Defect Site After Treatments

**Figure 7:**
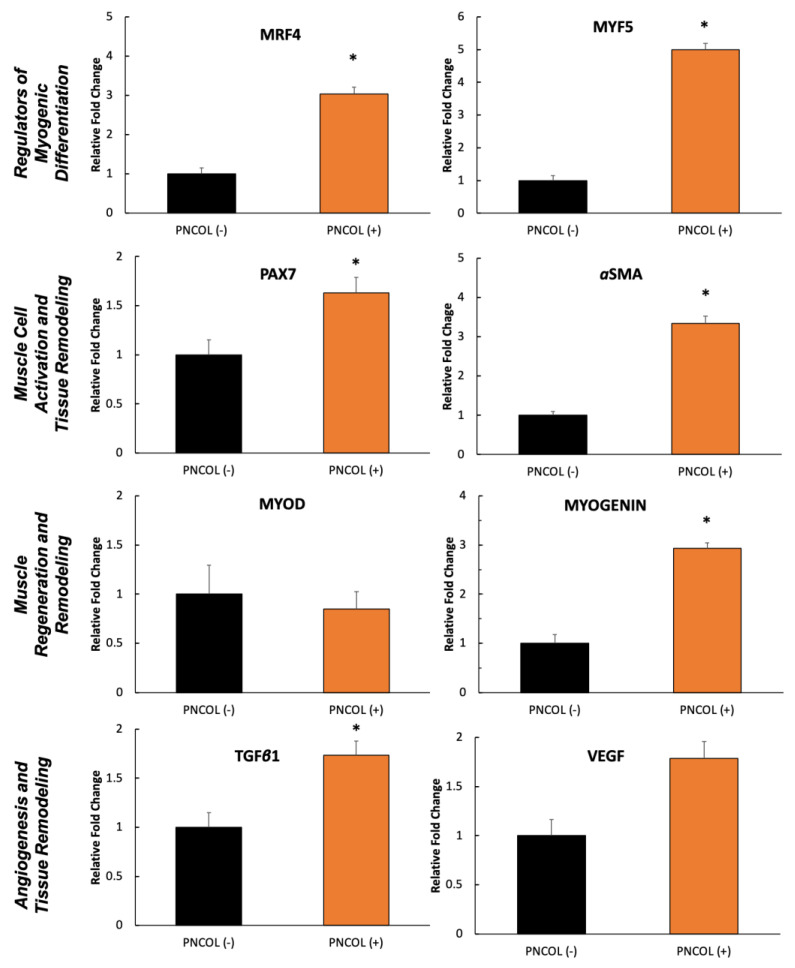
The relative fold changes of TA muscle markers of PNCOL (−), and PNCOL (+) samples, following 7 days of organ culture in a dynamic mechanical environment. All data were expressed as the mean and standard errors. * indicates the statistical differences between groups with *p* < 0.05, n = 4; at least three technical replicates were used for gene expression analysis.

To understand the effect of mechanical stimulation and PNCOL treatment on muscle regeneration at the molecular level, a comprehensive gene expression analysis was conducted on PNCOL-treated and PNCOL (−) samples in all replicated experiments. Figure 7 shows the relative gene expression fold change in important muscle markers between PNCOL (−) and PNCOL (+) groups following 7 days of organ culturing. MRF4 and MYF5, genes encoding transcription factors crucial for regulating skeletal muscle development, were upregulated 3-fold and 5-fold, respectively, in the PNCOL (+) groups compared to PNCOL (−) groups. Also, PAX7 and αSMA, genes that play distinct but complementary roles in maintaining muscle repair and tissue remodeling, were upregulated in the PNCOL (+) groups (Figure 7). Of the two important genes, myod and myogenin, which orchestrate the muscle cells’ differentiation into mature muscle fibers, only myogenin demonstrated an upregulation of almost 1.5-fold after PNCOL treatment. Finally, the expression of the VEGFA and TGFβ1 genes was upregulated almost 2-fold in the PNCOL (+) group compared to the PNCOL (−) group. The roles of VEGFA and TGFβ1 are critical in promoting angiogenesis and regulating inflammatory and fibrotic responses.

**Figure 8:**
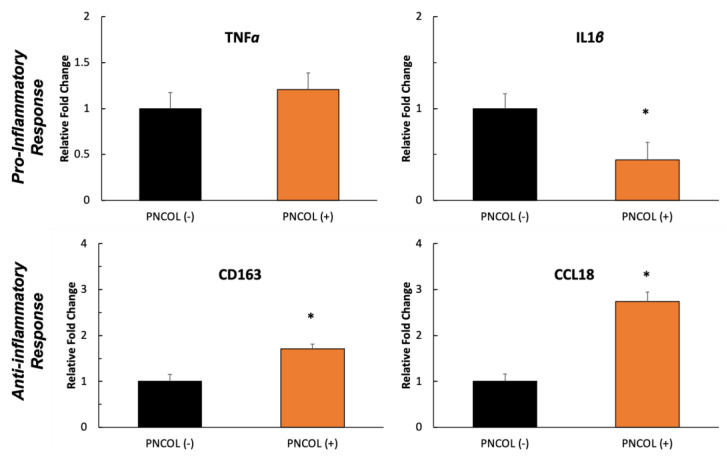
The relative fold changes of the inflammatory response of PNCOL (−) and PNCOL (+) samples following 7 days of organ culture in a dynamic mechanical environment. All data were expressed as the mean and standard errors. * indicates the statistical differences between groups with *p* < 0.05, n = 4; at least three technical replicates were used for gene expression analysis.

Next, genes involved in the inflammatory response were analyzed. As shown in Figure 8, TNFα and IL1β showed no significant difference between the PNCOL (+) and PNCOL (–) groups. However, the CD163 and CCL18 genes, associated with anti-inflammatory and tissue remodeling processes, were upregulated 1.5-fold and 3-fold, respectively, in the PNCOL (+) samples compared to the PNCOL (–) counterparts. These results indicate the potential efficacy of PNCOL in promoting muscle tissue repair and mitigating inflammation under mechanical loading in an ex vivo organ culturing platform.

### 2.7. Inflammation Stability of TA Muscle Defect Site After Treatments

**Figure 9:**
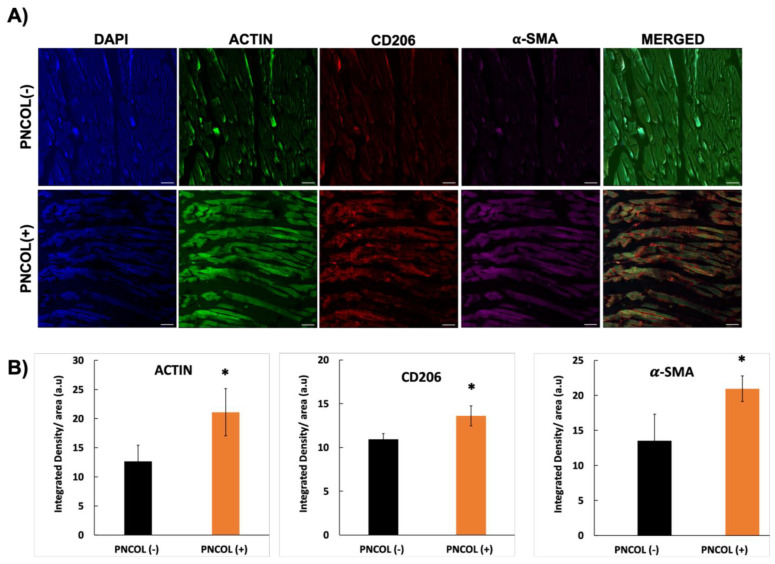
(**A**) Antibody staining of the PNCOL (−) and PNCOL (+) groups after 7 days of mechanical stimulation. Phalloidin (green) for f-actin staining, CD206 (red) as an anti-inflammatory marker, αSMA (magenta) for muscle regeneration marker, and DAPI (blue) for cell nuclei. (n = 4). Images at a 20× magnification; the scale bar represents 100 µm. (**B**) Quantification of actin, CD206, and α-SMA signal intensities normalized to the tissue area for the PNCOL (−) and PNCOL (+) groups. Bars represent mean ± SD (n = 4). * indicates significant difference compared with control (*p < 0.05).

Finally, to elucidate the impact of PNCOL treatment on TA defect under mechanical stimulation at the molecular level, protein expression via immunostaining was conducted. Figure 9 shows the presence of CD206 and αSMA surface markers in the PNCOL (+) compared to the PNCOL (–) group along with the quantitative analysis result.

CD206, a marker associated with anti-inflammatory processes and tissue remodeling, and αSMA, a marker indicative of fibrotic response, reflect the modulation of immune responses and fibrotic reaction towards enhanced muscle regeneration in the PNCOL (+) group compared to the control. The changes in actin in the PNCOL (+) samples were observed and compared with the PNCOL (–) group. Figure 9 demonstrates a more dominant presence of actin in the PNCOL (+) group compared to the PNCOL (–) group, as seen in all 20× figures and later confirmed in the zoom-in figures (63×). This relevant presence of actin in the PNCOL (+) group may indicate enhanced cytoskeletal rearrangement and cell migration, suggesting that PNCOL treatment could promote more efficient tissue repair processes through modulation of actin dynamics.

Quantitative image analysis further revealed that PNCOL treatment significantly increased the expression of structural, pro-healing macrophage, and contractile markers compared to untreated controls. The actin intensity increased from 12.6 ± 2.7 for the PNCOL (–) group to 21.1 ± 4.0 a.u. in the PNCOL (+) group (*p* < 0.05). Similarly, CD206 intensity, representing pro-resolving macrophage activity, increased from 10.9 ± 0.6 to 13.62 ± 1.4 (*p* < 0.05). The α-SMA intensity, a marker of early myofiber remodeling and contractile alignment, rose from 13.5 ± 3.8 to 20.91 ± 1.8 (*p* < 0.05). These results demonstrate that PNCOL enhances both cytoskeletal organization and regenerative macrophage polarization within the defect region.

## 3. Discussion

Recognizing the significance of whole organ culture in replicating complex physiological environments is necessary for advancing our understanding of tissue response and enhancing therapeutic outcomes. Thus, in this study, not only is an innovative ex vivo hindlimb dynamic organ culturing platform introduced, but also, the role of an injectable cell-laden nanofibrous matrix in critical-size defect TA muscle regeneration was studied under physiologically relevant mechanical loading. Before extensive biological and structural analyses, we first aimed to demonstrate the transmission of mechanical loading applied to the organ throughout its structure, by characterizing the bone structure. In ex vivo organ culturing, upon mechanical loading, notably, the cortical bone of the tibia and femur exhibited increases in total area and marrow area, without changes in cortical bone mass, which can be interpreted as a result of increased periosteal bone formation, where bone and muscle are juxtaposed, with simultaneously increased bone resorption at the endosteal surface, which may indicate a heightened pro-inflammatory environment in the absence of blood perfusion and innervation. The concept of muscle and bone being intertwined in their functions and physiology suggests that changes in one tissue can significantly impact the other [51]. These, together with demonstrating that cell viability and hindlimb integrity are maintained throughout the experiment, reassure the efficacy of the ex vivo hindlimb organ culturing platform.

The optical images (days 0 and 7) of the hindlimbs cultured using a dynamic organ culture platform demonstrated the gross health of the samples throughout treatments (Figure 2A). The cell viability, assessed using the colorimetric-XTT assay, remained preserved throughout the 7-day organ culture period for both PNCOL (−) and PNCOL (+) samples (Figure 2B). Importantly, there was no statistical difference in cell viability between day 1 and day 7 for either group, suggesting that the ex vivo mouse hindlimb culturing platform, irrespective of PNCOL treatment, maintains consistent cell viability over the culturing period. Overall, the µ-CT images and image-based quantifications (Figure 1), the optical images, and cell viability data (Figure 2) suggest that the mouse hindlimb organ culturing platform under dynamic mechanical loading conditions can transmit the mechanical loading from the muscle to the bone, as experienced in locomotion, while preserving the viability of the organ. Thus, the ex vivo mouse hindlimb organ culturing platform used in this study offers a valuable tool for investigating the isolated effects of variables such as mechanical loading, biomatrix for bone or muscle regeneration, electrical stimulation, ultrasound treatment, and more. Unlike in vivo studies, this platform provides a controlled environment that minimizes costs and enhances consistency. By isolating various factors that may influence outcomes, researchers can gain deeper insights into tissue responses without the complexities inherent in in vivo studies.

Once the viability of the ex vivo mouse hindlimb organ culturing platform was established, a detailed analysis was conducted to understand the potential of the myoblast-laden injectable matrix (PNCOL) in TA muscle regeneration under dynamic loading of 12% with 0.1 Hz frequency within the ex vivo mouse hindlimb organ culturing platform. The histological images of the damaged site (Figure 3) demonstrated that the TA defect treated with PNCOL had a high number of nuclei (purple dots within H&E images in Figure 3) corresponding to the presence of satellite cells and higher muscle regeneration [52,53]. Histological and quantitative analyses demonstrated that PNCOL treatment statistically significantly enhanced tissue regeneration within the critical-sized TA defect. The increased G-trichrome-positive areas in PNCOL-treated samples indicate improved collagen matrix organization, suggesting that PNCOL provides a favorable microenvironment for synchronized extracellular matrix remodeling.

The PNCOL (+) group regenerated the muscle histomorphology at the defect site. The normalized proteoglycan content data (Figure 4) agreed with the histological data, suggesting that GAG content increased statistically significantly more than 3-fold in TA treated with PNCOL (+) over the organ culturing period. Overall, the histological data, along with the GAG content data, suggested that there was a successful de novo extracellular matrix (ECM) regeneration following the injury for the TA muscle treated with the PNCOL. The newly generated ECM fibers at the defect site were visualized with SEM (Figure 5). The SEM micrographs showed that the gap at the defect site was woven with the ECM fibers in the PNCOL (+) groups, while there was a visible gap for the PNCOL (−) group after 7 days.

Following structural and quantitative analyses of the ECM component, proteomic and gene expression analyses were conducted to understand the cellular and subcellular level changes leading to higher ECM production for the PNCOL (+) groups. Cytokine and chemokine production was analyzed through the media of individual ex vivo organ cultures after 7 days. For instance, the higher expression of adiponectin in the PNCOL (+) group seen in the cytokine profile after the treatments might describe a favorable shift toward regeneration. The role of adiponectin appears to have a direct impact on muscle tissue regeneration [54]. The cytokine profile demonstrated an increased expression of CXCL1, CXCL2, and CXCL16 chemokines regulating inflammation and immune cell recruitment, which are crucial processes in tissue repair and regeneration. These chemokines are implicated in orchestrating the early stages of neutrophil, macrophage, and other immune cell infiltration into injured muscle tissue, thereby facilitating muscle regeneration [55,56]. Also, insulin-like growth factor-binding proteins (IGFBPs) regulate bone metabolism and play a crucial role in modulating the bioavailability and activity of IGFs, which are regulators of bone growth and remodeling [57]. IGFBP5 and IGFBP6 demonstrated a higher expression in the PNCOL (+) groups compared to the untreated counterparts. The intricate balance between IGFBPs influences bone formation and resorption processes during the crosstalk between muscle, bone, and endocrine factors in maintaining musculoskeletal health [57]. Overall, the cytokine and chemokine profile array demonstrated a positive response to both PNCOL and mechanical stimulation in 7 days of ex vivo organ culturing. Since the media extracted from each culture may have other components and smaller no-present proteins were not expressed on our proteome profile, gene expression analysis was conducted to thoroughly identify the regenerative capabilities of the bone–muscle organ after the treatments.

The relative gene expression of α-SMA, PAX7, and myogenin genes was increased significantly in the PNCOL (+) group in comparison to the PNCOL (−) group. α-SMA is an actin expressed by myofibroblasts, and the increased presence of αSMA denotes that myofibroblasts incorporated with the PNCOL have survived and proliferated [58,59]. Similarly, MYOD is a vital regulator of skeletal muscle metabolism, and no significant difference in expression was found between the PNCOL (+) and PNCOL (–) groups. In tandem with myofibroblasts’ increased survival and proliferation, the stagnating MYOD expression is consistent. Muscle cell metabolism regulation remaining unchanged for a larger population of cells allows more cells to survive [60]. The PAX7 gene is uniformly expressed by satellite cells in human muscle tissue and is a significant component in the regenerative properties of muscle tissue [61,62]. Moreover, Figure 7 contains the gene expression of Mrf4, MYF5, and TGFB1, which significantly increased expression in the PNCOL (+) group, while VEGFA remained constant. Mrf4 functions to negatively regulate MSK growth, whereas MYF5 functions to promote growth [63]. These two genes enforce homeostasis between the promotion and restriction of muscle growth. Though increased expression of Mrf4 alludes to hindered muscle growth, the greater expression of protein MYF5 relative to the increase in Mrf4 shows the presence of increased regeneration. TGFB1 belongs to the protein family of transformative growth factors and promotes the proliferation of MSK cells [64]. Additionally, VEGFA functions as a regulator for various endothelial cell activities such as proliferation and migration. Though endothelial tissue is irrelevant in this study, the role of VEGFA is to act as a diagnostic for the overall health of the tissue. The increase in Mrf4, MYF5, and TGFB1 supports the idea of bolstered muscle regeneration within the PNCOL (+) group [65,66]. A similar expression of VEGFA between the two groups suggests the continued health of the tissue.

The inflammatory response during muscle regeneration coordinates the clearing of damaged tissue, recruitment of immune cells, and initiation of satellite cell activation, crucial steps that support the repair and remodeling of muscle fibers [53,67]. Figure 8 demonstrates pro- and anti-inflammatory (TNFα, IL1β, CD163, and CCL18) gene expression. TNFα functions as a regulator of the inflammatory response and expressed no statistical difference between the PNCOL (+) and PNCOL (−) groups. Instead, a minor increase in expression in the PNCOL (+) group was seen; this is likely the product of the increased presence of muscle regeneration factors in the PNCOL (+) group. IL1β is a cytokine interleukin and is integral to the tissue-bound inflammatory response [68]. Most notably, IL1β exacerbates cell-wide damage during the inflammatory response greatly, yet not the regenerative factors associated with the inflammation [69]. CD163 is a macrophage-based protein and is an additive marker for tissues responding to inflammation [70]. CCL18 is a scientifically ambiguous protein and may have functions of chronic inflammation [71]. The fluctuations in protein expressions across the PNCOL (+) and PNCOL (−) groups support the idea of PNCOL causing an overall larger inflammation response than the control. CCL18 is expressed at a much more significant fold change, and though IL1β and CD163 present an inconsistency, the respective decreases and increases in each protein are not significant relative to the increase in CCL18.

This suggestion is further supported by immunostaining qualitative analysis (Figure 9A) with increased tissue regeneration and the presence of anti-inflammatory markers. The actin expression and localization can offer valuable information about the progression and efficacy of the regeneration process in musculoskeletal tissues [59,72,73]. The quantitative analyses (Figure 9B) of immunofluorescence images further confirmed this fact. The increased fluorescence intensity of actin, CD206, and α-SMA in PNCOL-treated tissues indicates a coordinated enhancement of structural integrity, immunomodulation, and myofiber remodeling within the critical-sized defect. Elevated actin expression reflects improved cytoskeletal organization and cellular alignment, while upregulation of CD206 suggests a shift toward an anti-inflammatory, pro-healing macrophage phenotype that promotes tissue repair. Concurrent elevation in α-SMA implies active matrix remodeling and early contractile fiber regeneration. Together, these findings demonstrate that PNCOL not only accelerates structural regeneration but also promotes a favorable regenerative microenvironment that supports functional tissue remodeling in skeletal muscle repair.

Overall, the data demonstrates that the ex vivo organ culturing platform can keep the bone and muscle tissue functions over 7 days of the culturing period under mechanical loading conditions. In addition, the data proves that the critical-size defect in TA muscle can be treated with PNCOL injection to the defect site. Our study underscores the efficacy of PNCOL treatment combined with 3D mechanical loading using an ex vivo organ culturing platform for promoting muscle tissue regeneration and reducing inflammation. These findings demonstrate the importance of multidimensional approaches for enhancing therapeutic outcomes in MSK disorders.

In addition, drawing on recent debates in the field of bioethics, this study also develops an interdisciplinary approach and adheres to the 4Rs principle (Reduction, Refinement, Replacement and Responsibility) for ethical animal research [74,75] through employing an ex vivo approach with isolated mouse hindlimbs instead of in vivo testing. Our motivation aligns with the growing emphasis on minimizing the number of animals used in research [76] and developing a robust sense of responsible animal experimentation.

It is important to note that while the ex vivo hindlimb dynamic organ culturing platform, along with the cell-laden injectable PNCOL matrix, offers valuable insights into muscle tissue regeneration, this study has some limitations as well. For instance, its scalability to larger animals may be limited. This platform may primarily be applicable to mice and rats, which exhibit notable differences in muscle regeneration compared to humans. The ex vivo model has inherent limitations. For instance, once the limb is harvested, it is no longer perfused by blood circulation or innervated by the central nervous system. This may inherently create an ischemic condition, where oxygen and nutrients must diffuse into tissues from the culture medium, and metabolic waste may accumulate over time. These factors likely limit the duration for which the limb can remain fully healthy. While our results show that the limb tissues can be maintained alive for at least 7 days, longer culture periods might require perfusion systems or vascular supplementation to prevent hypoxia and nutrient depletion. For future investigation, immunostaining for HIF1a can reveal whether the ex vivo-cultured mouse hindlimbs are differentially oxygenated compared to in vivo counterparts. Additionally, the state of the individual cell types within the cultured muscle–bone organ, including muscle fibers, bone, and endothelium, warrants further investigation. For instance, immunohistochemistry or single-cell RNA sequencing can be incorporated to further characterize the specific cell populations at the defect site to distinguish the mixture of different cell types, including satellite cells. For example, confirming PAX7 upregulation at the protein level could be achieved by immunostaining for Pax7-positive satellite cells in the TA muscle sections, and verifying myogenic differentiation could involve staining for developmental myosin heavy chain or other muscle fiber markers in the defect area.

## 4. Methods and Materials

### 4.1. Ex Vivo Organ Culture Platform and Mouse Hindlimb Culturing Under Mechanical Loading

**Figure 10:**
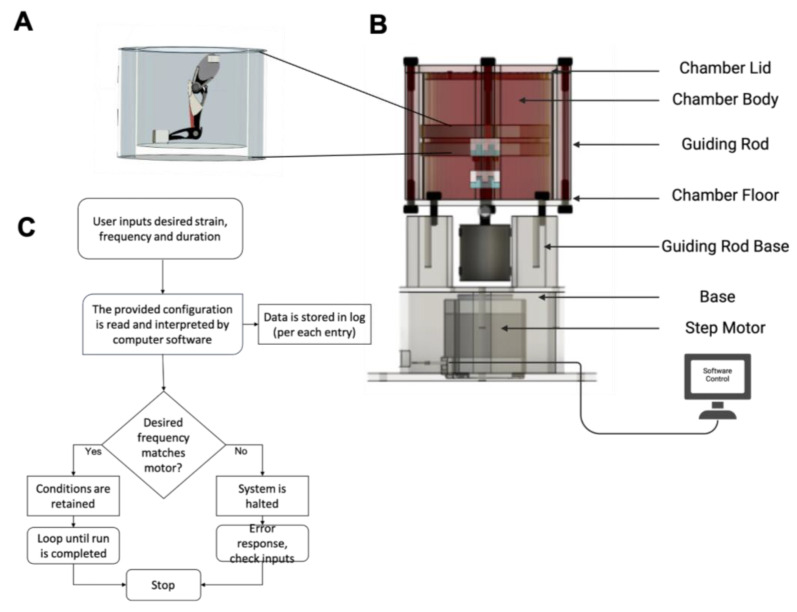
Organ culture platform components and system flowchart. (**A**) A schematic representation of mouse hindlimb within the biocompatible organ culturing chamber. (**B**) Components of the organ culturing platform. (**C**) Architectural and functional schematic of the hardware and software of the organ culture platform.

The organ culture platform, an innovative, custom-built mechanical loading platform, was designed to create physiologically relevant mechanical stimuli for the whole bone–muscle organ. Unlike other mechanical loading platforms, our proposed design creates predefined uniform mechanical strain on the whole organ while maintaining the stability of the tissues. The organ culture platform (Figure 10) is designed to load the entire bone–muscle organ onto the platform through fixed grips at the top and bottom ends. The uniaxial tension of the organ produces mechanical strain in the bone and muscle tissues. This gives us control over the movement of the entire organ structure and control over the amount and duration of strain applied, simulating different physiologically relevant environments. The uniaxial tensile organ culture platform’s working mechanism detailed in this protocol provides mechanical stimulation through a stepper motor displacement. This type of stimulus has been widely used to simulate heart muscle and musculoskeletal tissues, including tendon, ligament, bone, and cartilage, through cyclic tensile force [77,78,79]. Figure 10 shows the schematic of the ex vivo hindlimb organ culturing platform with mechanical loading.

As shown in Figure 10A, the chamber is an elliptical cylinder made up of biocompatible resin, a biologically compatible material (Formlabs, Somerville, MA, USA). The chamber lid and chamber floor seal the longitudinal ends of the chamber. The chamber is restricted in axial movement using guide rods. The guiding rods are affixed to the guiding rod base via threading and stainless steel screws, and the guiding rod base is affixed to the base identically. The aperture in the guiding rod base allows for the protrusion and retraction of the stepper motor, which moves vertically and provides tensile stress to the legs in the chamber. Through a wiring system, the organ culture platform is connected to a computer interface to control the platform. The sample is attached via stainless steel clamps and an adhesive from the lid to the floor of the organ culture platform (Figure 10A). The functional map shown below in Figure 10C provides the hardware and software logic of the organ culture platform.

**Ex Vivo Mouse Hindlimb Culturing:** To test the efficacy of the organ culturing platform, ex vivo mouse hindlimbs culturing was conducted under physiologically relevant mechanical stimulation for 2 h a day for 7 days. The ex vivo mouse hindlimbs were subjected to 12% uniaxial tensile strain at a frequency of 0.1 Hz to replicate the dynamic physiological conditions of MSK tissues, including tendon, ligament, and muscle. The 12% uniaxial strain corresponds to the stress encountered by skeletal tissues, mirroring the strain experienced during exercise, which can reach up to 12.5 times body weight. This strain also reflects the length changes of 5–9% observed during moderate physical activities and up to 12% during high-intensity exercise [59]. A strain magnitude of 10–15% closely approximates the physiological stretch range experienced by skeletal muscles, such as the tibialis anterior during locomotion and high-intensity activity [23]. Multiple studies including ours [26,59,79,80,81] have demonstrated that cyclic mechanical loading at or above 12% strain and at 0.1 Hz promotes tissue remodeling and muscle regeneration without inducing tissue damage [82,83,84]. For instance, Frey et al. specifically demonstrated that C2C12 myoblasts respond robustly to 12% cyclic strain [85]. Chan et al. also demonstrated that 15% stretch at 0.1 Hz enhanced myogenic markers in engineered muscle constructs with C2C12 cells [86]. Taken together, 12% strain at 0.1 Hz is both physiologically relevant and effective at promoting mechanotransduction and tissue formation without damaging the tissues and the cells.

For ex vivo mouse hindlimb organ culture, hindlimbs were collected from 8-month-old wild-type male mice (C57BL/6 strain) that had expired prior to receipt by the research team. No animals were euthanized or sacrificed specifically for this study, consistent with the principles of Reduction and Refinement. Tissue isolation and de-skinning were performed immediately post-mortem to preserve tissue integrity and viability. All animal care and husbandry procedures (protocol #107229) complied with the NIH National Research Council’s Guide for the Care and Use of Laboratory Animals and were reviewed and approved by the University of Toledo Health Science Campus Institutional Animal Care and Use Committee (IACUC). The University of Toledo animal facility is AAALAC-accredited and operates as a pathogen-free facility.

These relatively young, physically mature male mice are analogous to young, physically fit athletes, susceptible to muscle injury [87,88,89]. All dissection for hindlimb procedures followed protocols approved by the Institutional Animal Care and Use Committee and the University of Toledo. Briefly, the abdomen was then cut and separated using blunt-end scissors, and surface muscles were removed to expose the pelvic hip joint. The pelvic hip joints of both hindlimbs were then cut, and the hindlimbs were removed from the abdomen. The skin and hair of each hindlimb were removed with blunt dissection. Then, hindlimbs were placed on ice in phosphate-buffered saline solution (PBS) for 2 h. The hindlimbs were then placed into an organ culture platform with DMEM media supplemented with FBS 10%, 1% P/S, 1:500 Primocin, 1.5% 0.4 M NaCl, and 1.5% 0.4 M KCl for culturing for 7 days.

### 4.2. Micro-CT Analysis of Mouse Hindlimb After Ex Vivo Organ Culturing Under Mechanical Loading

Assessment of cortical bone in the tibia and femur was conducted by micro-CT using the µCT 35 system (Scanco Medical AG, Bruettisellen, Switzerland). Bone scans were performed with the X-ray source operating at 70 kVp and 114 µA energy settings, recording 500 projections/180° acquired at a 300 ms integration time using a 7 µm nominal resolution voxel for both bone locations. Scans of the cortical bone at the tibia and femur midshaft contained 57 slices, all of which were contoured automatically and segmented at a 260 per mille threshold. Simulated bone bending strength (I_max_/C_max_ and I_min_/C_min_) and torsional strength (pMOI) at the tibia midshaft were based on bone cross-sectional geometry in combination with local tissue mineral content [90]. Analysis of the trabecular bone microstructure, cortical bone parameters, and simulated bone strength was conducted using the Evaluation Program V6.5-3 (Scanco Medical AG) and conformed to recommended guidelines [91].

### 4.3. Myoblast Cell-Laden Injectable Matrix Synthesis for TA Muscle Regeneration

#### 4.3.1. Cell-Laden Injectable Nanofibrous Matrix for Tibialis Anterior Muscle Regeneration

Cell-based injectable tissue scaffolds play a pivotal role in tissue regeneration by providing a supportive framework for cellular growth and organization for restoring damaged tissues. To regenerate damaged tibialis anterior muscle, an injectable and nanofibrous called the PNCOL matrix was synthesized based on our well-established protocols [48,92,93].

**Figure 11:**
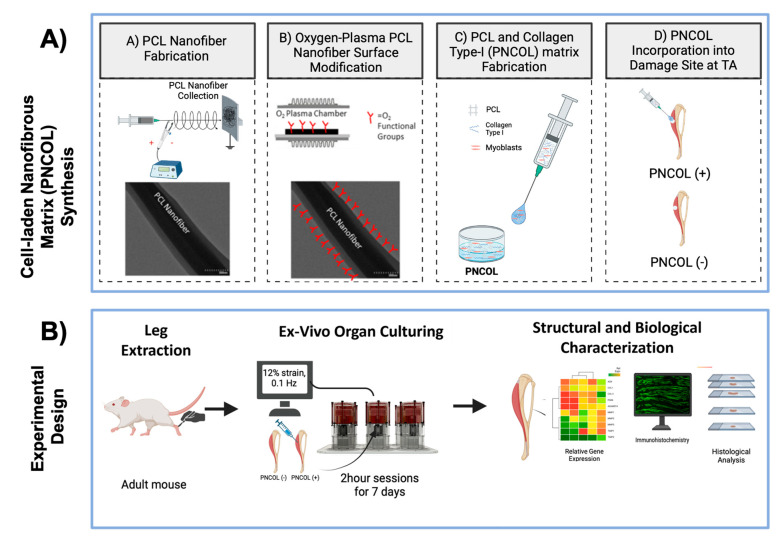
(**A**) Myoblast cell-laden PNCOL (polycaprolactone and collagen type-I) matrix synthesis to be injected at tibialis anterior (TA) muscle defect. (**B**) The major steps followed in ex vivo organ culture experimental design: isolation of adult mouse hindlimbs; PNCOL incorporation at tibialis anterior (TA) defect site; cyclic loading at 12% strain, I 0.1 Hz for 2 h/day over 7 days, post-culture structural and biological analyses including gene expression, immunohistochemistry, and histology.

Briefly, PCL (MW 45,000, Sigma-Aldrich, St. Louis, MI, USA) pellets were dissolved overnight in a 3:1 mixture of chloroform and methanol solution under a sterilized chemical hood at 16% *w*/*v*. The following day, the solution was loaded into a syringe pump and extruded at a flow rate of 8 mL/h, through a 20-gauge needle. A 20 kV potential was applied between the needle tip and a collector plate, where PCL nanofibers were collected (Figure 11A). Following 2 h of PCL nanofiber collection, the PCL fibers were transferred under the chemical hood for residual solvent evaporation overnight. The following day, the PCL nanofibers were homogenized using a high-speed homogenizer (Ultra-Turrax, IKA Works, Inc., Staufen, Germany) and functionalized using oxygen-plasma treatment for 3 min to reduce its hydrophobicity based on our prior studies [48,92,93]. Upon incubation, the PCL nanofibers were mixed within the neutralized collagen type-I solution with a 3% (*w*/*v*) concentration (Figure 11A).

The neutralized collagen type-I solution with 3 mg/mL concentration was prepared from 9.1 mg/mL collagen type-I stock solution (Corning, Corning, NY, USA) with a pH of ~3.4 using 1 M NaOH, phosphate-buffered solution (PBS) and culture media. Then, the mouse myoblasts cell line (C2C12, ATCC, USA), cultured in a complete media of Dulbecco’s Modified Eagle’s medium (DMEM) (ATCC; Manassas, VA, USA), supplemented with 10% fetal bovine serum (FBS) (Corning; USA) and 1% penicillin–streptomycin (Corning; USA), was encapsulated within the PNCOL with a cell density of 10^6^ cells/mL (Figure 11A).

#### 4.3.2. TA Muscle Defect Creation and Cell-Laden Matrix Injection to the Defect Site

Following extraction, critical-size creation of muscle loss injury was performed on the tibialis anterior (TA) muscle using a 3 mm biopsy punch (Robbins Instruments Chatham, Chatham, NJ, USA) on each ex vivo mouse hindlimb. In the skeletal muscle literature, a critical-sized or volumetric muscle loss (VML) defect is defined as an injury involving approximately ≥ 20% of the total muscle mass, which exceeds the tissue’s endogenous regenerative capacity and therefore fails to spontaneously regenerate [94,95,96]. Full-thickness TA defects produced by 2–2.5 mm punches, corresponding to ~20% mass excision, are widely recognized as VML or critical-sized injuries and consistently display hallmark non-regenerative pathology, including fibrosis and limited myofiber regeneration, in the absence of treatment [97,98]. Size-thresholding studies in murine limb musculature have further identified 3 mm defects as explicitly critical [47,99]. Accordingly, the 3 mm TA punch employed in this study lies at or above the established critical-size threshold in murine models.

Following defect creation, 200 μL of the myoblast-laden nanofibrous matrix, called PNCOL, was injected into the defect areas. Hindlimbs in which the defect site received no PNCOL treatment served as a control. Then, all defect sites were sutured using the modified purse-string suture techniques (MPSSs) based on our established protocol [50]. Two overlapping suture loops were interconnected and contracted circumferentially for each defect site to provide near-watertight sealing. Following suturing, all sample groups were moved to the organ culture chamber for 12% mechanical strain for 2 h/day for 7 days.

### 4.4. Assessing the Effect of Cell-Laden Injectable Matrix for TA Muscle Regeneration Following Ex Vivo Organ Culture Under Dynamic Mechanical Loading

TA muscle regeneration was characterized using structural and biological assessment including gross defect morphology, histology, and antibody staining. Defect morphology was measured by photographing and weighing the experimental and control groups before and after the 7 days. Post healing, 5 μm long longitudinal samples were retrieved roughly 400 μm deep in the damaged area. These samples were used in concurrence with hematoxylin and eosin and G-trichrome stains to identify inflammation around the PNCOL-injected area (damage site). Gene expression, cytokine production, and protein analysis were utilized to assess the regenerative effect of the treatments at the damage site (TA muscle). Figure 11A demonstrates a schematic representation of the cell-laden injectable PNCOL matrix synthesis process, while Figure 11B demonstrates the major steps followed in ex vivo organ culture experimental design.

#### 4.4.1. Structural and Morphological Characterizations of the Ex Vivo Mouse Hindlimb Organ Culturing Under Mechanical Loading

(1) Scanning Electron Microscopy Analysis

TA muscle, PNCOL matrix, and collagen fiber morphology were characterized using Scanning Electron Microscopy (SEM) (Hitachi, Santa Clara, CA, USA) after ex vivo organ culturing with mechanical stimulation. Before characterization, TA from the damaged site was cut and fixed with 4% paraformaldehyde in PBS for 30 min. After fixation, the samples were dehydrated first in sequential ethanol solutions, with increasing concentrations from 30% to 100% for 15 min each. The samples were then further dehydrated by being submerged into sequential ethanol/hexamethyldisilane (HMDS) solutions from 30% to 100% for 10 min each to improve optical image quality. After the chemical treatment, the samples were left to air-dry overnight. The gold-sputter coating was applied to the samples the following day, allowing for visualization under SEM to observe structural and morphological changes upon experimental setup.

(2) Histological Analysis and Quantification of Histological Data

Muscle tissue samples extracted from the damage site of the PNCOL (−) and PNCOL (+) groups were submerged in 10% normalized formalin for 48 h. The tissue samples were then processed using a Sakura Tissue TEK VIP 5 (Torrance, CA, USA) tissue processor, dehydrating the samples in 70%, 85%, 95%, and 100% ethanol before being treated in a clearing agent (FISHER XS-05 laboratory-grade Xylene). After dehydration treatment, samples were sealed in paraffin (Leica Paraplast Plus, Richmond, IL, USA) and implanted into a tissue cassette before being incised on a LEICA RM 2235 microtome (Rankin Biomedical, Davisburg, MI, USA) in 5 mm sections and placed on slides. The slides were incubated in hematoxylin and eosin (H&E) and G-trichrome staining, washed, and viewed under a bright field microscope.

Quantitative analysis of histological images was performed using ImageJ software version 1.54p (NIH, Bethesda, MD, USA). For each specimen, high-resolution images obtained under identical microscope settings were converted to 8-bit grayscale, and color thresholds were applied to isolate G-trichrome-positive (blue) regions. The thresholding parameters were standardized across all samples to ensure consistent detection. The positive staining area was measured and normalized to the total tissue area in each image to calculate the percentage of G-trichrome-positive regions. Data from each sample (n = 4 per group) were averaged to generate group means ± standard deviation.

#### 4.4.2. Biological Characterization of the Ex Vivo Mouse Hindlimb Organ Culturing Under Mechanical Loading

(1) Cell Viability Analysis

Cell viability on the mouse hindlimb culture was assessed on days 1 and 7 using the XTT cell viability assay kit (Sigma Millipore, Product No. 11465015001, Saint Louis, MO USA). This assay measures metabolic activity as an indicator of cell viability, specifically focusing on mitochondrial enzyme activity. Formazan production, directly proportional to the number of metabolically active cells, occurs when XTT is reduced in viable cell mitochondria. The absorbance of the resulting colored solution correlates with cell viability, determined by comparing samples. Triplicates of 100 μL of media extracted from each organ culture chamber were incubated on a 96-well plate and mixed with 70 μL of XTT assay reagent. Following an 8 h incubation at 37 °C, the wells’ optical density (OD) was measured using a UV kinetic microplate reader at 450 nm, with a reference wavelength of 660 nm. Each plate included appropriate blank control wells containing media without reagent. Absorbance readings were calculated according to the vendor’s protocol and extrapolated to the initial time point.

(2) Proteome Array Analysis

The Proteome Profiler Mouse XL Cytokine Array kit (R&D Systems, Minneapolis, MN, USA) was used to analyze cytokine, chemokine, and growth factors released from whole hindlimbs in culture after 7 days of mechanical stimulation and PNCOL treatment. Each kit provided four membranes, sufficient for four distinct samples. The membranes featured 111 capture antibodies in duplicate, targeting various biomolecules as listed on the manufacturer’s website. Conditioned media samples (1–2 mL) were collected and stored on ice during collection after 7 days of treatment (mechanical stimulation and PNCOL) and then stored at −80 °C until further use. Upon thawing, samples underwent centrifugation at 2000 rpm for 3 min to remove cellular debris. The supernatant, devoid of cellular components, was reserved for experimentation and maintained on ice throughout the process; experimental procedures adhered to the protocol outlined by the manufacturer. Image analysis was conducted using HLImage++ software version 6.2 (Western Vision Software, Salt Lake City, UT, USA ) to interpret results. Cytokines with pixel density (PD) differences greater than 200 pixels between groups were selected. This threshold method facilitates the criteria aimed at identifying biomarkers indicative of significant changes in expression levels.

(3) Gene Expression Analysis

The TA muscles (damage site) from PNCOL (+) and PNCOL (−) groups were extracted and dissected after experiment termination for gene expression analysis. Gene expression was carried out using real-time polymerase chain reaction (qRT-PCR). Tissue samples were extracted one day after the experiment’s termination and prepared by snap freezing and mechanical disruption. RNA was isolated using a Directo-zol RNA Extraction Kit (Zymo Research, Irvine, CA, USA). Per the manufacturer’s instructions, this isolated RNA was reverse-transcribed using the Superscript IV Reverse Transcriptase (ThermoFisher, Plainville, MA, USA). Quantitative real-time PCR was performed using the iTaq Universal SYBR Green Supermix (Bio-Rad, Hercules, CA, USA) to investigate the expression of muscle regeneration markers and immune response markers. The relative gene expression for fold changes between PNCOL (−) and PNCOL (+) samples was obtained using the ∆∆Ct method. The ∆∆Ct method utilizes glyceraldehyde-3-phosphate dehydrogenase (GAPDH) as a housekeeping standardizing gene. RT-qPCR was performed in the iCycler iQ detection system (Bio-Rad, Hercules, CA, USA), with thermocycling for 35 cycles. The primer sequences were obtained from the published literature and were designed and purchased from Integrated DNA Technologies (IDT, Coralville, IA, USA). The genes and associated primer sequences are listed in Table 1.

(4) Immunostaining and Immunohistochemical Analysis

Building upon the gene expression analysis, further evaluation of the immune response following treatments involved assessing the TA muscle tissue at the damage site through immunostaining. Paraffin-embedded samples were sectioned into ~40 um thick slices using a microtome (Reichert- Jung, Model 820 II, Buffalo, NY, USA), mounted on glass slides, and cleared using a xylene/ethanol rehydration protocol. Following deparaffinization and rehydration, the slides were incubated in trypsin and calcium chloride solution (0.1% (*v*/*v*) trypsin (ATCC, USA) plus 0.1% (*w*/*v*) calcium chloride (Sigma-Aldrich, USA) at 37 °C for 30 min. Then, heat-mediated antigen retrieval was performed by immersing the slides in citrate buffer (10 nM sodium citrate in 0.02% Tween 20) at 95 °C for 30 min. For immunostaining, samples were permeabilized with 0.1% Triton x-100 in PBS. Then, sections were washed and blocked in 2.5% goat serum and 0.2% Tween in PBS for 20 min. After blocking, slides were incubated overnight at 4 °C in mouse anti-Smooth Muscle Actin (1:40) (AB262054, Santa Cruz, CA, USA) and recombinant rabbit CD206 antibody (1:40) (ThermoFisher, MA1-35936). The next day, slides were washed with PBS and 0.2% Tween solution and blocked in 2.5% (*v*/*v*) goat serum for 20 min. Then, slides were incubated with Alexa Four 488 Phalloidin (1:200) (ThermoFisher, A12379), goat anti-rabbit IgG (H + L) Highly Cross-Absorbed Secondary Antibody Alexa Flour 594 (1:200) (ThermoFisher, A32740) and Goat anti-Mouse IgG (H + L) Highly Cross-Absorbed Secondary Antibody, Alexa Flour 657 (ThermoFisher, A32728TR) for 2 h. Finally, slides were incubated with DAPI (4’,6-Diamidino-2-Phenylindole) (Life Technologies, Waltham, MA, USA) dye (1:1000) in PBS for 30 min and washed with 0.2% Tween. Slides were then imaged using an HC PL APO 63X/1.40 OIL CS2 or HC PL FLUOTAR L 20× air objective with a Leica Stellaris 5 confocal system equipped with HyD detectors and the LASX version 5.3.1 software. Confocal images from at least three independent studies were processed to visualize each cell’s nuclei, filamentous actin (F-actin), αSMA, and CD206 expressions.

Quantitative immunofluorescence analysis for actin, CD206, and α-SMA was performed using ImageJ (NIH, Bethesda, MD, USA). For each specimen, the background subtraction was applied, followed by measurement of Integrated Density for each marker. The mean intensity was normalized to the total tissue area to obtain the Integrated Density per Area (a.u.) for each marker. Data from each sample (n = 4 per group) were averaged to generate group means ± standard deviation.

### 4.5. Statistical Analysis

All statistical analyses were conducted using R-Studio (version 2024.02). Data are presented as mean ± standard deviation (SD) unless otherwise specified. Normality of data distribution was verified using the Shapiro–Wilk test, and equality of variances was confirmed using Levene’s test prior to applying parametric tests. For single-variable comparisons between two independent groups (PNCOL (–) vs. PNCOL (+)), an unpaired two-tailed Student’s t-test was used when normality was met; otherwise, a Mann–Whitney U test was applied. For datasets involving repeated measures or multiple variables (e.g., gene expression panels), a two-way ANOVA followed by Benjamini–Hochberg false discovery rate (FDR) correction was performed to account for multiple comparisons. Each biological experiment used n = 4 independent biological replicates (hindlimbs from four animals), and each assay included at least three technical replicates per sample to ensure reproducibility. This sample size is consistent with prior ex vivo organ culture studies and was selected to minimize animal use while maintaining adequate statistical power. Statistical significance was defined as *p* < 0.05. (*) indicates difference between control and treated groups (*p* < 0.05), while (#) indicates the difference between same-day experimental groups after multiple-comparison adjustment.

## 5. Conclusions

This study presents a novel ex vivo mouse hindlimb organ culture system as a physiologically relevant and ethically conscious platform for investigating skeletal muscle regeneration following critical-size injury. Unlike conventional in vitro models, this approach preserves the native musculoskeletal architecture—including muscle, bone, vasculature, and connective tissues—allowing for spatially and mechanically integrated responses to be studied in a controlled environment without systemic confounders. The platform was used to evaluate the effect of the injectable, cell-laden nanofibrous collagen scaffold (PNCOL) on the regeneration of a tibialis anterior muscle defect. Our system replicated physiological strain conditions (12% at 0.1 Hz), simulating loading patterns encountered during musculoskeletal activity. The combination of cyclic strain and PNCOL delivery promoted myofiber regeneration and a shift toward anti-inflammatory macrophage polarization, as confirmed through immunohistochemistry, cytokine profiling, and gene expression analysis. This work not only highlights the therapeutic potential of cell-laden nanofibrous collagen scaffold-based interventions but also demonstrates the feasibility and translational relevance of an ex vivo whole-limb organ culture model. The integration of biomechanical loading, bioengineered scaffolds, and intact organ physiology in a single ex vivo system represents a significant advancement in musculoskeletal research and may serve as a model for future studies in muscle repair, mechanotransduction, and immunomodulation.

## Figures and Tables

**Table 1:** Forward and reverse primers for real-time PCR.

Gene	Forward Primer	Reverse Primer	Ref.
MRF4	5′-GCAAGACCTGCAAGAGAAC-3′	5′-GCGAAAGGAGGAGGCTTAA-3′	[60]
MYF5	5′-CCGTGTTTCCCATGGTTGTG-3′	5′-GAGCACTCGGCTAATCGAAC-3′	[60]
PAX7	5′-CAGCCAACTGTGATCCTGCT-3′	5′-CTTCATATGCGGCATCCACG-3′	[60]
αSMA	5′-GTCAGCACTTCGCATCAAGG-3′	5′-TTCACAGGATTCTGGGAGCGG-3′	[58]
MYOD	5′-GCAGGTGTAACCGTAACC-3′	5′-ACGTACAAATTCCCTGTAGC-3′	[60]
MYOGENIN	5′-GCCACAGATGCCACTACTTC-3′	5′-CAACTTCAGCACAGGAGACC-3′	[60]
TGFβ1	5′-GGTTATCTTTTGATGTCACCG-3′	5′-GTTATGCTGGTTGTACAGGG-3′	[100]
VEGF	5′-GGTGCATTGGAGCCTTGCCT-3′	5′-TGGTGAGGTTTGATCCGCAT-3′	[100]
CD163	5′-TCTGTTGGCCATTTTCGTCG-3′	5′-TGGTGGACTAAGTTCTCTCCTCTTGA-3′	[100]
CCL18	5′-AAGAGCTCTGCTGCCTCGTCTA-3′	5′-CCCTCAGGCATTCAGCTTAC-3′	[100]
TNFα	5′-AGAGGGAAGAGTTCCCCAGGGAC-3′	5′-TGAGTCGGTCACCCTTCTCCAG-3′	[100]
IL1β	5′-CCAGCTACGAATCTCGGACCACC-3′	5′-AGCAATGGTAAACCAGTAGTTGG-3′	[100]
GAPDH	5′-GGCATTGGTCTCAATGACAA-3′	5′-TGTGAGGGAGATGCTCAGTC-3′	[100]

## Data Availability

The original contributions presented in this study are included in the article. Further inquiries can be directed to the corresponding author.

## Abbreviations

CCL-18	Chemokine (C-C motif) ligand-18
CD163	Cluster of Differentiation 163
CD206	Cluster of Differentiation 206
IFN-γ	Interferon gamma
ECM	Extracellular matrix
FBS	Fetal bovine serum
GAPDH	Glyceraldehyde-3-phosphate dehydrogenase
IL	Interleukin
IL1β	Interleukin beta 1
LPS	Lipid polysaccharide
VEGFA	Vascular endothelial growth factor A
TGFB1	Transforming growth factor beta 1
MMP	Matrix metalloproteinase
MRF4	Myogenic regulatory factor 4
MYF5	Myogenic regulatory factor 5
PBS	Phosphate-buffered saline
PD	Pixel density
PCR	Polymerase chain reaction
PNCOL	Polycaprolactone nanofiber and collagen
qRT-PCR	Quantitative real-time polymerase chain reaction
RNA	Ribonucleic acid
RPMI	Roswell Park Memorial Institute
TNF-α	Tumor necrosis factor-α
C2C12	Immortalized mouse myoblast cell line
List of Units and Symbols:	
M	Molar
U	Unit
*v*/*v*	Volume per volume
*w*/*v*	Weight per volume
μ	Micro
